# Immunogenic Cell Death-Based Cancer Vaccines

**DOI:** 10.3389/fimmu.2021.697964

**Published:** 2021-05-31

**Authors:** Ming-Zhu Jin, Xi-Peng Wang

**Affiliations:** Department of Gynecology and Obstetrics, Xinhua Hospital Affiliated to Shanghai Jiao Tong University School of Medicine, Shanghai, China

**Keywords:** cancer vaccine, danger associated molecular patterns, immunogenic cell deaths, immunotherapy, tumor microenvironment

## Abstract

Cancer immunotherapy has achieved great advancement in the past decades. Whereas, its response is largely limited in immunologically cold tumors, in an urgent need to be solve. In recent years, an increasing number of studies have shown that inducing immunogenic cell deaths (ICDs) is an attractive approach to activate antitumor immunity. Upon specific stress, cancer cells undergo ICDs and dying cancer cells release danger associated molecular patterns (DAMPs), produce neoantigens and trigger adaptive immunity. ICDs exert a cancer vaccine-like effect and Inducement of ICDs mimics process of cancer vaccination. In this review, we propose a concept of ICD-based cancer vaccines and summarize sources of ICD-based cancer vaccines and their challenges, which may broaden the understandings of ICD and cancer vaccines in cancer immunotherapy.

## Introduction

Cancer treatment has shifted from tumor cell-centric to tumor microenvironment (TME)-centric with an in-depth understanding of the constitution and function of TME (1). Meanwhile, apart from targeting oncogenic pathways, there is an increasing awareness of the relationship between tumorigenesis and immunity. In the past decades, cancer immunotherapy has revolutionized cancer treatment, with immune checkpoint blockades (ICBs) including programmed cell death protein 1 (PD-1)/programmed cell death protein-ligand 1 (PD-L1)/cytotoxic T lymphocyte associate protein-4 (CTLA-4) being regarded as one of the most promising approaches to treat cancer. However, ICBs do not fit all types of tumor. They encounter unresponsiveness in “cold” tumor (glioblastoma, ovarian cancer, prostate cancer, etc.), characterized by the lack of tumor antigens, T cell priming, activation or infiltration (2). Impaired T cell priming or activation was attributed to defective recruitment of antigen presenting cells (APCs) and lack of co-stimulatory molecules including danger associated molecular patterns (DAMPs) (3). Cancer vaccines are such a strategy which turns “cold” tumor microenvironment to “hot” one assisting to solve the therapeutic resistance of cancer immunotherapy.

Immunogenic cell death (ICD) is a unique cell death featured by activation of dying T cell immune response and release of DAMPs. The most frequent applied DAMPs to evaluate the immunogenicity of cell death including calreticulin (CRT), adenosine triphosphate (ATP) and high mobility group box 1 (HMGB1). DAMPs induced by tumor cell ICD, as mentioned before, are critical to maturation of dendritic cells (DCs), production of immunosuppressive factors, activation of APCs and T cell co-stimulation (2, 4, 5). The characteristics of ICD provide a theoretical basis for it to be utilized to produce cancer vaccine.

Bonaventura and colleagues have provided an elaborate summary of approaches to turn “cold” tumors into “hot” ones (2). They also highlighted that ICDs induced by chemotherapy or radiotherapy and cancer vaccines are potential approaches to activate and recruit T cells (2). Herein, we will demonstrate the significance of cancer vaccines in cancer immunotherapy, highlight that ICDs can be targets for cancer vaccines and summarize current strategies of inducing ICDs. We believe that this review will provide an updated, deep and comprehensive understanding of ICD-based cancer vaccines and lead the way for cancer immunotherapy.

## Current Status of Cancer Immunotherapy

Thirty-five years have passed when interferon-α (IFN-α), the first cancer immunotherapy, was approved by FDA for treating hairy cell leukemia (6). The concept of cancer immunotherapy has shifted from enhancement to normalization of antitumor immunity (7). Great progress have been made in ICBs, cancer vaccines, chimeric antigen receptor T (CAR-T) cells therapies, natural killer (NK) Cells therapies, co-stimulatory receptor agonists and cytokines in the past several decades. For example, myeloid cell TREM2 reprogrammed tumor microenvironement and the anti-TREM2 treatment promoted responsiveness of anti-PD-1 immunotherapy (8, 9). Personalized ovarian cancer vaccine produced neoantigen-specific T cells and prolonged survival of OC-bearing mice (10). CAR-NK therapy received great therapeutic responses in 11 enrolled relapsed or refractory CD19-positive cancers, without inducing major immune related adverse effects (irAE) (11). Cancer therapy has gradually entered into the era of immunotherapy. Currently, PD-1/PD-L1 monotherapies or combination therapies have been approved by FDA for first-line therapy of patients with metastatic gastric cancer and esophageal adenocarcinoma cancers, advanced renal cell carcinomas, triple-negative breast cancers, advanced lung cancer or advanced head and neck cancers. Past 5 years have even witnessed appear of neoadjuvant ICBs, which were believed to stimulate a long-lasting immunomodulatory effects on diverse immune cells. Over 100 registered clinical trials on neoadjuvant ICBs were recruiting, undergoing or completed. However, despite the rapid development, cancer immunotherapy do face some challenges need to be solved. Hegde and Chen have summarized top 10 challenges for immunotherapy, including the demand in the advancement in pre-clinical models, further explorations in mechanisms of cancer immunity, assessment of its clinical efficacy, as well as investigation of its combination regimens to improve therapeutic response or reduce irAE (12). Among which, we believe that transforming immunologically cold tumors into hot ones to maximize clinical efficacy of cancer immunotherapy/ICBs is one of the hottest research focuses.

## Cancer Vaccines

Cancer vaccines can be categories into genetic vaccines, protein or peptide vaccines and cell vaccines. In the past decade, the recognization of cancer vaccines in immunotherapy gradually increase, especially in its combination with ICBs. Some have already been used in clinical practice. Human papillomavirus, Hepatitis B Virus and Hepatitis C virus vaccines are applied to prevent oncogenic infections, also called prophylactic vaccine. Oncophage, a heat shock protein (HSP) vaccine, was approved in Russia for patients with earlier stage kidney cancer in 2008 (13). Sipuleucel-T (Provenge^®^) was the first United States (U.S.) Food and Drug Administration (FDA)-approved cancer vaccine (2010), used to treat metastatic prostate cancer. It was a cancer vaccine manufactured with peripheral-blood mononuclear cells and a recombinant protein PA2024 through *ex vivo* incubation. Sipuleucel-T was reported to prolong overall survival of patients with metastatic prostate cancer, but did not delay disease progression (14). Oncophage and Sipuleucel-T are typical examples of therapeutic cancer vaccines, whose clinical realization is quite limited owing to time and cost taken to generate personalized cancer vaccine. Regardless of cancer vaccines formats, cancer vaccines aim at enhancing immunogenicity and promoting antitumor immunity to eliminate cancer cells through the induction of cancer antigens.

Cancer antigens can be classified into tumor associated antigens (TAAs), tumor specific antigens (TSAs) and cancer germline antigens (14). Non-mutant TAAs are suggested to be targets for cancer vaccines. Recently, Sahin and colleagues have tested the effect of FixVac, a liposomal RNA (RNA-LPX) vaccine that targets four non-mutated TAAs in ICB-experienced melanoma. The study showed that the RNA-LPX vaccine alone or combined with ICBs enhanced antitumor immunity, resulted in a better therapeutic response and confirmed that TAAs-based cancer vaccine could be utilized in immunotherapy (15). Whereas, there are still concerns that TAAs-based cancer vaccines encounter therapeutic resistance (central tolerance) as TAAs are generally expressed in normal cells. TSAs, also known as neoantigens, which are derived from tumor specific mutations rather than normal cells. It is believed that neoantigen-based cancer vaccines may be safer, more effective and more likely to spare from resistance, bringing cancer vaccine back to research focus after two decades when cancer vaccine clinical trials encountered a low objective response rate (16). Classified by sources, classical neoantigen-based cancer vaccines include but not limited to synthetic long peptide (SLP) vaccines, DC vaccines, RNA vaccines. In a phase I/Ib surgical resectable methylguanine methyltransferase-unmethylated glioblastoma trial, Keskin and colleagues have shown that SLP vaccination do lead to neoantigen-specific T cell infiltration and response in tumor microenvironment, which sensitizes immunotherapy in tumors that originally with low tumor burden and immunogenicity (17, 18). Though, unfortunately all the enrolled patients could not spare from death because of tumor progression and recurrence (18), TSA-based cancer vaccines do arouse great attention. There is an urgent need for broaden understandings of vaccine-induced TSAs in stimulating immunity and improvements in manufacturing TSA-based cancer vaccines.

## Inducing Immunogenic Cell Death to Generate Cancer Vaccination

### Immunogenic Cell Death: A Form of Immunogenic Regulated Cell Death

ICDs, contrary to tolerogenic or non-immunogenic cell deaths, are a form of regulated cell deaths that trigger adaptive immunity through production of neoantigens and release of DAMPs and cytokines. Thus, immunogenicity of cancer cell deaths *in vitro* is evaluated by immunostimulatory DAMPs like HMGB1, ATP, CRT, HSP70, HSP90, ANXA1, or cytokines like IFN, CCL2, CXCL1, CXCL10, etc (19). Meanwhile, the vaccination-rechallenge model is applied to detect ICDs *in vivo*. ICDs were initially found to be triggered by chemotherapy or radiation, while accumulative evidence has shown that they can be induced by photodynamic therapy (PDT), targeted therapy, oncolytic viruses, cardiac glycosides, as well as shikonin and capsaicin, extracts of Chinese herbal medicine. The inducement of ICDs and the production of neoantigens reshapes immunosuppressive TME to immunoactivited one, initiate antitumor immunity and improve therapeutic response of immunotherapy. ICD-based therapy is now viewed as attractive candidates for cancer immunotherapy combination regimens.

### ICD Induction Realizes Cancer Vaccine-Like Effect

**Figure 1:**
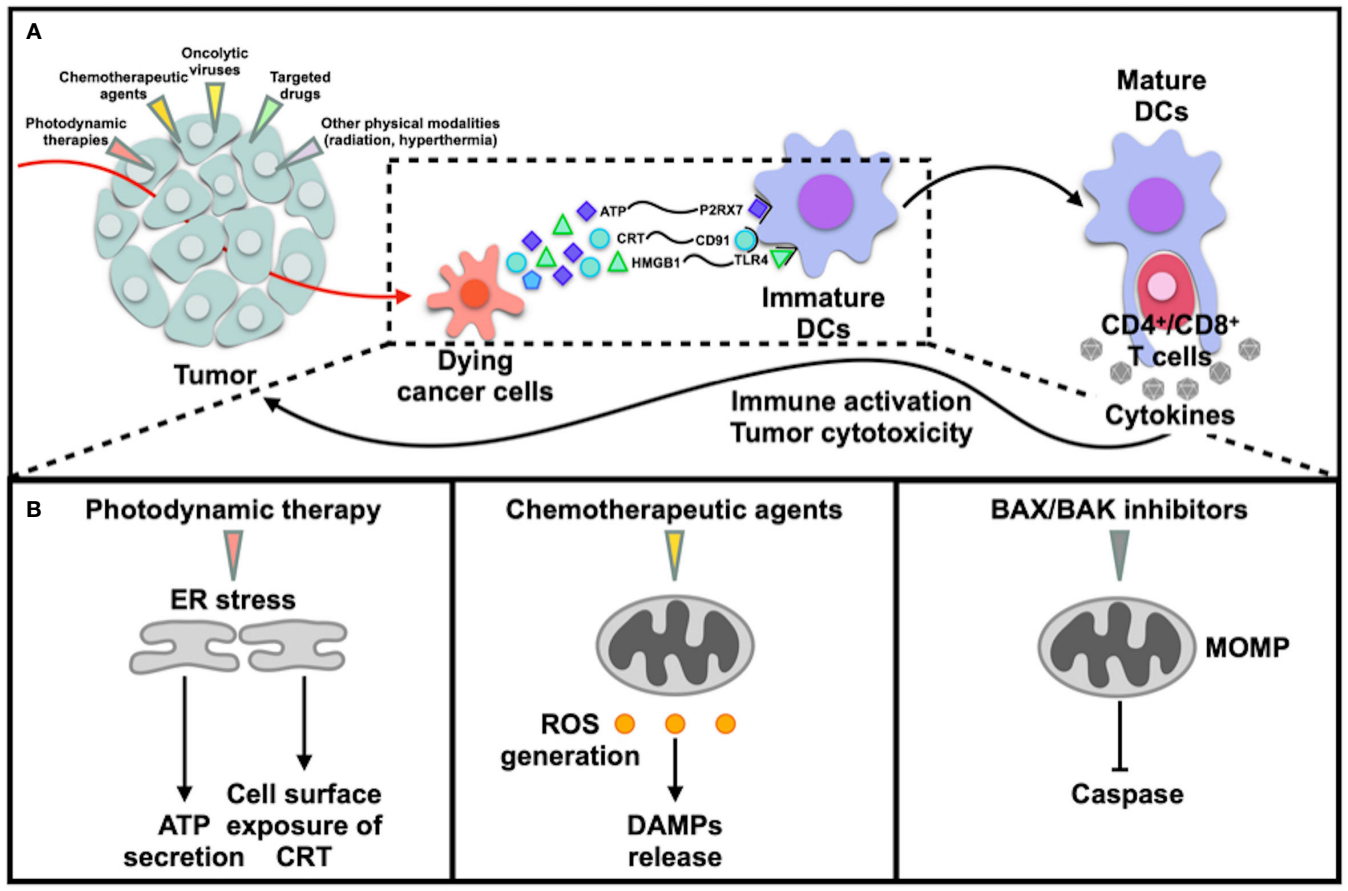
Immunogenic cancer death. **(A)** Various triggers like photodynamic therapies, chemotherapeutic agents, oncolytic viruses, targeted drugs and physical modalities induce immunogenic cell deaths and release of DAMPs, leading to immune activation; **(B)** ICDs were induced via different mechanisms: 1) induction of ER stress directly cause cell deaths; 2) generation of ROS, in which induction of ER stress does not directly cause ICDs; 3) induction of mitochondrial outer membrane permeabilization (MOMP).

Upon particular stress like chemotherapy, radiotherapy, ICD cascade initiates including generation of tumor specific neoantigens by dying cancer cells, generation of ROS, induction of ER stress, release of DAMPs including exposure of CRT on cell surface (activation of “eat me” signal), secretion of ATP and HMGB1. DAMPs are then recognized by pattern recognition receptors (PRRs), promoting recruitment and maturation of DCs (**Figure 1**). The mature DCs then uptake dying cells, process TSAs to APCs, promote T cells polarizations, engulf CTLs, CD4^+^ T cells, which leads to production of cytokines and activation of antitumor immunity ultimately (4, 5) (**Figure 1**). During the process, dying cancer cells generate antigens and enhance immunogenicity to kill tumor cells, in line with the criterion of endogenous cancer vaccines. Recruited DCs may serve as be effective targets for cancer vaccine as well, which is DC vaccine that we are familiar with.

### ICD Detection Relies on the Vaccination-Rechallenge Experiment

**Figure 2:**
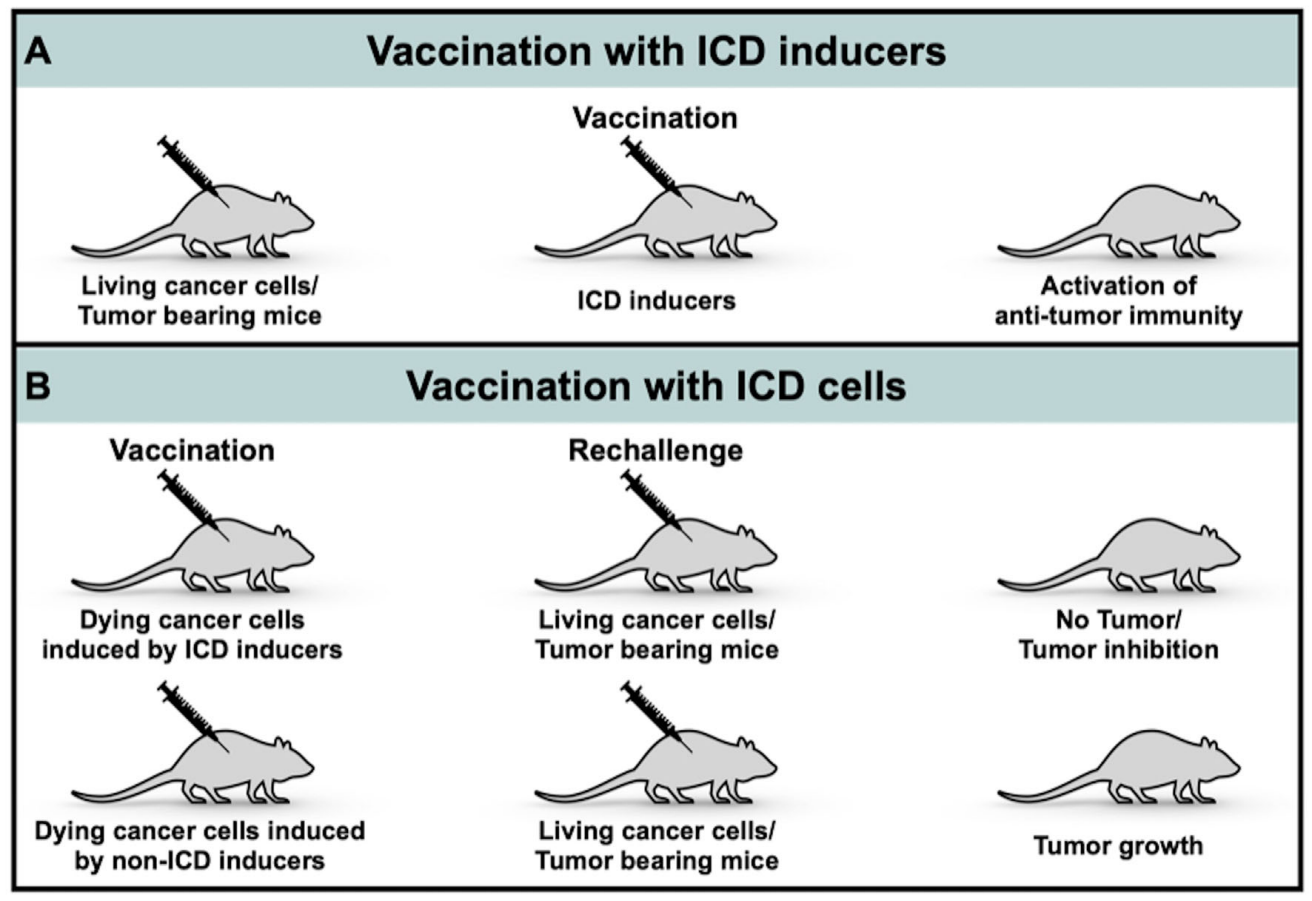
Process of inducing ICD-based cancer vaccines. **(A)** Vaccination with ICD inducers; **(B)** Vaccination with ICD cells.

Immunogenicity of cell deaths induced by therapeutic agents can be assessed *in vivo* and *in vitro* through quantitative analysis of CRT exposure, ATP and HMGB1 secretion, ROS generation or ER stress. At present, there is no specific structure that helps to predict potential ICD inducers. The gold standard experiment to validate ICD or verify an ICD inducer is the vaccination-rechallenge experiment: cancer cells were treated with a potential ICD inducer and cell viability was assessed *via* cytometry after staining with PI and annexin V/DIOC_6_ (**Figure 2B**). Most of non-dead cancer cells should be at a dying stage. Cell suspension was then injected subcutaneously to immunocompetent mice, which is called vaccination. Living cancer cells were injected a week later, which is called rechallenge. Tumor volumes of mice post-vaccination to post-rechallenge and the ratio of mice spared from tumor formation were recorded (20). The vaccination-rechallenge experiment not only helps to identify an ICD inducer, but also builds a prototype for vaccination with ICD cells.

## Approaches to Produce Immunogenic Cell Death-Based Cancer Vaccines

ICD can be triggered by PDT (21), chemotherapeutic agents (18, 22–31), oncolytic viruses (OVs) (32–34) and targeted drugs (35–38) (**Table 1**), as well as physical modalities such as radiation (52), hyperthermia (53), which won’t be discussed herein. In this review, we divide ICD-based cancer vaccines into two categories: ICD inducers (**Figure 2A**) and ICD cells (**Figure 2B**).

**Table 1:** Examples of immunogenic cell death-based cancer vaccine.

Categories	Examples	Cell lines	Vaccination-challenge experiment	ROS generation	ER stress	Biomarkers (DAMPs) for identifying ICDs	Cytokines secretion	Antitumor immunity activation	Other observations	Type of ICDs	Ref
**Photodynamic therapies-derived cancer vaccine**	PS-PDT; PD-PDT; FAL-ICG-HAuNS; AuNC@MnO2, AM; Ce6/MLT@SAB; CCPS; pRNVs/HPPH/IND; Ds-sP/TCPP-TER NPs)	4T1; B16; CT-26; MC38; GL261; MCA205	+/-	+	+/not mentioned	CRT exposure; ATP release; HMGB1 release	IL-6; IL-12; TNF-α; IFN-γ	Recruitment, maturation and activated antigen presenting functions of DCs; Recruitment, infiltration, proliferation and activation of CTLs; Activation of CD4^+^ T cells (mostly Tregs) and NK cells activation; Inhibition of MDSCs proliferation	CHOP proteins upregulation; Relieve of hypoxic niche; Caspase-3 activation; PI3K-Akt pathway activation; IDO upregulation	Apoptosis; Ferroptosis; Necrosis	(21, 39–43)
**Chemotherapeutic agents-derived cancer vaccine**	Doxorubicin; Bortezomib; Melphalan; Paclitaxel	EG7; CT26; PROb; B16; U266; GL261;	+ (Mostly)	+	Not mentioned in most researches	CRT exposure; ATP release; HMGB1 release; HSP70 exposure; HSP90 exposure; F-actin exposure	IFN-γ; IL-1β; IL-6	Maturation and proliferation of DCs; Proliferation. infiltration of CTLs; Proliferation of NK cells; Proliferation and polarization of macrophages;	Caspase-3 activation; Activation of NF-kB–mediated CCL2 transcription; Activated vesicle exocytosis induced by IkappaB-mediated SNARE;	Apoptosis	(22, 24, 25, 27, 31, 44)
**Oncolytic viruses-derived cancer vaccine**	Semliki Forest virus; Vaccinia virus; Adenovirus; Measles virus; Coxsackievirus B3; Newcastle disease virus; Seasonal Influenza Vaccine	B16; HOS; A549; CT26; MeWo; PAN02; CMT93; Mel888; Mel624; SkMel28	+	+	Not mentioned in most researches	CRT exposure; ATP release; HMGB1 release; HSP90 exposure;	IFN-α; IFN-β; IFN-γ; IL-12; TNF-α; IL-4; IL-13; IL-6; IL-8; IL-1β; IL-24; CCL3	Activation and maturation of DCs; Recruitment and activation of tumor antigen-specific CTLs; Maintenance of Bregs;	Release of PAMPs; Activation of PI3K/Akt and MAP/ERK/MEK pathway; Release of tumor specific neoantigens	Apoptosis; Necroptosis; Pyroptosis; Autophagy;	(33, 34, 45–50)
**Targeted drugs-derived cancer vaccine**	7A7 mAb; Cetuximab; Crizotinib	D122; H1650;	+	Not mentioned	+	CRT exposure; ATP release; HMGB1 release; HSP 70 exposure; HSP 90 exposure; ANXA1 exposure;	IFN-α; IFN-γ; IL-12; IL-17;	Activation and maturation of DCs; Proliferation, infiltration and activation of CTLs; Promotion of DCs phagocytosis of dying cells; Induction of PD-1 expression on tumor-infiltrating CD4^+^ Foxp3^−^ cells	Inhibition of XBP1	Apoptosis	(35–37)
**ICD cells-derived cancer vaccine**	Necroptotic cancer cells generated by RIPK3 induction systems	CT26	+	Not mentioned	–	ATP release; LDH release; HMGB1 release;	IFN-γ; CXCL1	Activation of BMDCs maturation and phagocytosis of dying cells; Cross-priming and proliferation of CTLs	NF-kB activation, which is necessary for immunogenic necroptosis	Necroptosis	(51)

4T1, murine breast cancer cell lines; B16, murine melanoma cell lines; CT-26, MC38, murine colon carcinoma cell lines; GL261, murine glioma cell lines; MCA205, murine fibrosarcoma cell lines; U266, murine myeloma cell lines; ER, endoplasmic reticulum; CRT, calreticulin; DCs, dendritic cells; CTLs, cytotoxicity T lymphocytes; Bregs, regulatory B cells; Tregs, regulatory T cells; NK cells, natural killer cells; MDSCs, myeloid-derived suppressor cells; 7A7 mAb, an anti-murine EGFR Ab; RIPK3, receptor-interacting protein kinase-3; LDH, lactate dehydrogenase; XBP1, X-box binding protein 1; ANXA1, Annexin A1.

### Vaccination With ICD Inducers: Photodynamic Therapy

PDT has been proved to have anti-tumor and immune-activation effect in various types of cancers such as cervical cancer, pancreatic cancer, prostate cancer, glioblastoma and clinically approved in treating non-small cell lung cancers and esophageal cancers by the U.S. FDA. PDT requires a light-sensitive dye, also known as a photosensitizer (PS). With the presence of light of specific wavelength (600-900 nm) and oxygen, PDT is able to generate reactive oxygen species (ROS) and cause cell death through inducing endoplasmic reticulum (ER) stress (54, 55).

It is worth noting that PDT’s effect on inducing ICDs and enhance tumor immunogenicity is reduced in hypoxic niche (21, 56). Meanwhile, PDT exacerbates hypoxia in TME, leading to angiogenesis, tumor progression, metastasis, poor therapeutic response and prognosis (56, 57). Researchers have been trying to solve the problem by developing an oxygen nanocarrier with chlorine e6 encapsulated (C@HPOC) (58). The modified oxygen-boosted PDT displayed infiltration of cytotoxic T lymphocytes (CTLs) in TME and a better induction of ICD in 4T1 murine breast cancer cells by elevating cell surface exposure of CRT, increasing release of HMGB1 and ATP, and afterwards promoting the maturation of DCs (57). Combing metal-organic framework-based nanoparticles to PDT was also of benefit for alleviating PDT-induced hypoxia *via* inhibiting hypoxia inducible factor-1α (59). The combination of nanomaterials or anti-hypoxic treatment with PDT may help it to achieve a better clinical realization.

### Vaccination With ICD Inducers: Chemotherapeutic Agents

The initial hypothesis on the relationship between chemotherapy and ICDs could date back to one or two decades ago. It was proposed that the impact of chemotherapy on immune system through induction of ICDs helped it to achieve better clinical efficacy (18). However, not all kinds of chemotherapeutic agents induce ICDs, and some chemotherapeutic agents do have immunogenic effects, but insufficient to cause cell death (28). So far, anthracyclins (22), bleomycin (23), bortezomib (24), cyclophosphamide (25, 26), daunorubicin (22), doxorubicin (22), idarubicin (22), melphalan (27), oxaliplatin (31), paclitaxel (44) and trifluridine/tipiracil (29) have been proved to induce ICDs; while, cisplatin, mitomycin C were proved to induce non-immunogenic apoptosis (22, 28). The reason remains unknown, even though oxaliplatin and cisplatin share similar structures, which raises the bar for seeking out an ICD inducer and confirms the significance of vaccine-rechallenge experiment.

Mechanisms in inducing ICDs for chemotherapeutic agents are different from those for PDT. Instead of inducing ICDs directly through ER stress, chemotherapeutic agents tend to induce generation of ROS. Based on the difference, Wang et al. have divided ICD inducer into two types: type I for those inducing ROS generation (chemotherapeutic agents/targeted therapy), type II for those inducing ER stress (PDT/Oncolytic viruses) (60). Notably, cancer vaccination effects induced by chemotherapeutic agents are weaker than PDT. Take melphalan as an example, hypericin photodynamic therapy (Hyp‐PDT) as a positive control, brefeldin A (a tolerogenic cell death inducer) as a negative control, melphalan-induced cancer vaccination effect was in the middle with 40% vaccinated mice protected (62% for Hyp-PDT group, less than 20% for brefeldin A group) (27). However, apart from its cytotoxicity to kill tumor cells directly, chemotherapeutic agents have their advantages compared to ICD inducer in being a cancer vaccine: their extensive role on non-malignant cells in immunosuppressive TME and additional immunogenic effect other than ICDs, which may be utilized to realize better tumor-killing effect (30).

### Vaccination With ICD Inducers: Oncolytic Viruses

OVs have become attractive formats of cancer vaccine in immunotherapy considering its direct cytotoxicity mediated by oncolysis, immune activation and anti-angiogenesis (61). Even intratumoral injections of OVs have been proved to activate anti-tumor immunity not only inside tumors and on adjacent TME, but also on distant untreated focuses (62). Previous studies have revealed that oncolytic viruses such as Coxsackievirus B3, Adenovirus, Measles virus, Semliki Forest virus, Newcastle disease virus, influenza A virus elicit ICDs (33, 34, 45–47, 50) and improve immune responses of ICBs (48, 49). The additional release of pathogen-associated molecular patterns (PAMPs) derived from oncolytic viruses further increases immune-activation (63).

Specially, the combination of oncolytic viruses and chemotherapeutic agents have been shown to maximize immunogenicity. For example, the combination of adenovirus and oxaliplatin, both demonstrated to be ICD inducers in colorectal cancer in previous studies (31, 47), leaded to reduced tumor growth and longer median survival CT26 (mouse rectal adenocarcinoma cell line)-bearing mice compared to adenovirus or oxaliplatin alone (64). Instead of systemic administration, researchers chose intratumoral injection *via* interventional radiologic techniques to peruse a high local concentration sufficient to cause ICDs and a relatively low systemic toxicity. Meanwhile, a replicating other than a non-replicating virus was required to overcome transient growth inhibitions and achieve sustained tumor suppressive activity; buffer for oxaliplatin needed to be carefully selected considering its impact on increasing cytotoxicity of oxaliplatin and reducing infectivity of adenovirus (64). These findings confirm synergistic effects of two ICD inducers and provide guidance when one single ICD inducer does not sufficient to provide a strong and long-lasting immune-activation.

### Vaccination With ICD Inducers: Targeted Drugs

Epidermal growth factor receptor (EGFR)-targeting mAb 7A7 or cetuximab and tyrosine kinase inhibitor crizotinib were reported to exert ICDs (35–38, 52). EGFR-targeting mAb 7A7 exert an effect on DCs, T cells and NK cells infiltration (65). The combination of targeted drugs and chemotherapy, for example cetuximab plus folinic acid+fluorouracil+irinotecan (FOLFIRI) or crizotinib plus cisplatin), resulted in a better antitumor efficacy (36–38). Cetuximab’s ability of inducing ICDs relies on EGFR mutational status and BRAF mutation. It failed to induce ICDs in human colon cancer cells (HT-30 cells lines) (KRAS^WT^) (37). These provide explanations for heterogeneous or limited therapeutic efficacy of some targeted drugs when applied as a single agent to some extent. Liu and colleagues also showed that crizotinib and cisplatin combination upregulated expression of PD-1/PD-L1 and improved therapeutic response to ICBs (37, 38). However, targeted therapy in inducing ICDs is still at a beginning stage calling for more attempts and scientific evidence.

### Vaccination With ICD Cells

Expect vaccination with ICD-inducers, vaccination with ICD cells also realizes a cancer vaccine-like effect, which mimics the establishment of vaccination-rechallenge model (**Figure 2B**). Immunocompetent mice were vaccinated with doxycycline-treated necroptotic DD_RIPK3 cells (51). Injected necroptotic cells leaded to the generation of TSAs, proliferation of CTLs, release of HMGB1, production of cytokines like CXCL_1_, IFN-γ and phenotypic maturation of bone marrow-derived DCs. Remarkably, NF-κB activation but not ER stress was observed in necroptotic cells, indicating immunogenicity of DD_RIPK3 cells was not principally mediated by ER stress, which was a little bit different from up-mentioned classical ICDs (51). Strategies that elevated RIPK3 expression were required since losses of RIPK3 expression exists in many types of cancer and genotypes of ICD cells largely restrict their immunogenicity (6, 51). Compared to ICD inducers, utilizing ICD cells is more able to produce personalized cancer vaccines, but more time-consuming and money-consuming.

## Discussion

ICDs generate tumor specific antigens, which serves as endogenous cancer vaccine targets. Recent years, especially last year, have witnessed a growing utilization of nanoparticles in delivering ICD inducers or amplifying cancer cell ICDs. Advantages of ICD-based cancer vaccines are quite obvious. First and foremost, they arouse immunogenicity for “cold” tumor and sensitize immunotherapy. Secondly, their immune stimulation activities reach to distant untreated lesions, which means intratumorally injection with a lower-dose may be chosen to avoid systemic cytotoxicity. Apart from up-mentioned a great variety of inducers, other therapeutic agents such as cardiac glycosides (66), non-steroidal anti-inflammatory drugs (NSAIDs) (67), dinaciclib (an experimental inhibitor of cyclin-dependent kinases) (68) displayed ability to induce ICDs as well.

However, it is still difficult to look for a candidate in a bundle of drugs. Recognitions of universality of ICD inducers, ICD biomarkers and platforms for efficient, time-saving, money-saving, high-throughput drug screening may accelerate drug discoveries. What’s more, we have noticed heterogeneous capability of activating anti-tumor immunity. As reviewed previously, TME is made up of various specialized microenvironments (1), and there is an intricate crosstalk between each other. Besides fighting against immunosuppressive TME, ICD-based cancer vaccine needs to overcome hypoxia as well to realize a stable and long-lasting effect. Jessup and colleagues have combined two ICD inducers to achieve better immune responses (64). Chen et al. (58) and Cai et al. (59) have worked on modifying the PS with nanoparticles or combining anti-hypoxia treatment to overcome hypoxia in TME as mentioned before. They have set good examples for magnifying clinical effect of ICD-based cancer vaccines. The clinical realization of ICD-based cancer vaccines relies on a deeper investigation of their mechanisms not merely on tumor immunity or immune microenvironment but also on the whole TME, and more attentions and attempts on combination therapy. It is noteworthy that previous studies haven’t take irAE into consideration. Questions like how severe are irAE of ICD-based cancer vaccines or what kinds of people tend to suffer from irAE call for answers. Also, we’ve noticed that ICDs include apoptosis, ferroptosis, necrosis, autophagy, which may exert a different impact on immunity (69), whereas, few studies take modes of cell deaths into considerations.

In general, the concept that ICD can be competitive cancer vaccine targets provides a solid theoretical basis for application of ICD-based cancer vaccine in “cold” tumor and broadens understandings and approaches for investigation of neoantigen-based cancer vaccine. Owing to immunogenicity induced by ICDs, ICD-based cancer vaccines will be appealing sensitizers for ICBs in the near future.

## Author Contributions

X-PW and M-ZJ conceived the paper. M-ZJ wrote the paper and developed the figures and tables. X-PW edited the paper. All authors contributed to the article and approved the submitted version.

## Funding

X-PW is supported by the National Natural Science Foundation of China (Nos. 81874103 and 81930064).

## Conflict of Interest

The authors declare that the research was conducted in the absence of any commercial or financial relationships that could be construed as a potential conflict of interest.
